# Exosome-Loaded Bioscaffolds for Spinal Cord Injuries: A Review

**DOI:** 10.1155/sci/8841129

**Published:** 2025-07-30

**Authors:** Ruilin Chen, Jian Zheng, Jie Hao, Yang Yang, Shaohu Xu, Feiyu Zhang, Feng Zhang, Yu Yao

**Affiliations:** ^1^School of Medicine, Nantong University, Nantong, Jiangsu Province 226001, China; ^2^Department of Orthopedics, Affiliated Hospital of Nantong University, Nantong, Jiangsu Province 226001, China; ^3^Department of Trauma Center, Affiliated Hospital of Nantong University, Nantong, Jiangsu Province 226001, China

**Keywords:** biological material, exosomes, inflammatory reaction, neural repair, spinal cord injury, stem cells

## Abstract

Exosomes are naturally occurring cellular products released by various cell types in the body. Their composition is similar to that of human tissues, which reduces the risk of immune rejection. As critical mediators of intercellular communication, exosomes transmit signals and information that regulate the physiological states of surrounding tissues. Depending on their cellular origin and molecular content, exosomes can either promote nerve regeneration and functional recovery at the site of spinal cord injury (SCI) or exacerbate the local injury microenvironment. However, as a cellular product, the composition and function of exosomes are affected by the type and state of the cells from which they originate, and thus, there may be specificity problems in treatment, such as the possible instability of the therapeutic effect, et cetera. Moreover, exosomes need to be further optimized in terms of their delivery and release strategies in order to improve the duration and stability of the therapeutic effect. Thus, a single therapy approach is often insufficient to effectively support nerve repair following SCI. Numerous studies have demonstrated that encapsulating exosomes within biomaterial scaffolds enhances their delivery and retention at the injury site, thereby improving their viability. This paper reviews the latest research on stem cell-derived exosomes and biomaterials in the context of SCI. It further explores the combined application of exosomes and biomaterial scaffolds in SCI treatment, while also addressing the associated challenges and future prospects.

## 1. Introduction

**Figure 1:**
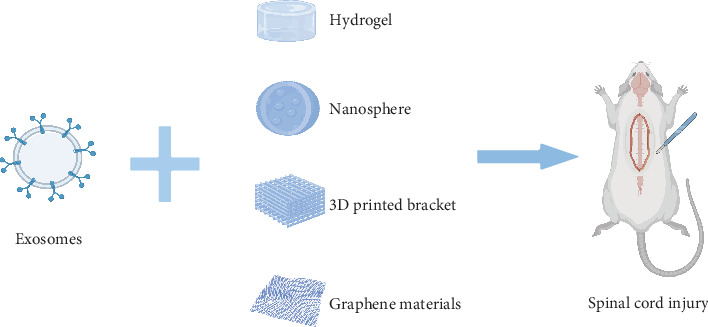
Exosomes derived from stem cells can be combined with hydrogels, nanospheres, 3D printed scaffolds, and graphene materials to treat spinal cord injuries, respectively. The image was produced by BioRender.com.

The spinal cord is part of the body's nervous system, located within the spinal column, and is responsible for transmitting signals from the brain to all parts of the body and transmitting sensory signals from all parts of the body back to the brain. The damage that can be caused to the spinal cord or its surrounding tissues when suffering from an external injury such as a car accident, fall, sports injury, or violence is known as a spinal cord injury (SCI). When the spinal cord is damaged, the signaling process is affected, leading to a variety of dysfunctions. The incidence of SCI is estimated to be between 250,000–500,000 per year globally [1]. The mainstay of treatment is surgical decompression and early surgical decompression has been shown to help reduce nerve damage after SCI [2]. In addition, steroid hormones are commonly used in clinical practice because of their powerful anti-inflammatory effects (inhibition of the production of inflammatory factors TNF-α, interleukin (IL)-1β, and IL-α), which can protect against increased inflammation at the site of SCI [3]. At the same time, the increased risk of infection associated with the use of high doses of steroid hormones is also worth pondering [4]. There have been many experimental advances in cell transplantation as an emerging therapeutic approach [5]. The cells typically transplanted are mesenchymal stem cells (MSCs), neural stem cells, oligodendrocyte progenitors, Schwann cells, and olfactory sheath cells [6]. Compared to cells, exosomes provide numerous benefits, such as reduced immunogenicity, simplified storage and transportation, neuroprotective capabilities, and the potential to traverse the blood–cerebrospinal fluid barrier [7]. The composition and release of the exosome can be influenced by the modulation of cell type and state [8], allowing its action in vivo to be precisely regulated and controlled, thereby improving the efficacy and safety of therapy. With the development of tissue engineering techniques, encapsulation of exosome on biomaterial scaffolds allows better transfer of exosomes to the site of injury and promotes regeneration of residual neuronal axons (Figure 1). In addition, biomaterial scaffolds need to have good biocompatibility, suitable biodegradability, and low immunogenicity [9]. Biomaterials used in SCI research can be categorized into natural materials, synthetic materials, composites, and micro- and nanomaterials. The primary materials currently utilized include hydrogels, nanomaterials, and three-dimensional (3D)-printed materials [10]. This review explores the present research landscape of stem cell-derived exosomes and biomaterials in the context of SCI. It discusses the integration of exosomes with biomaterial scaffolds for therapeutic purposes and examines the challenges and future prospects in this field.

## 2. Injuries to the Spinal Cord Pathophysiology

SCI is characterized by two pathological processes: primary and secondary injury. Primary injury refers to the immediate mechanical damage caused by external forces, such as compression, stretching, or puncture, which can result in fractures or dislocations of the spinal vertebrae. Secondary injury encompasses a series of delayed pathological events—including inflammation, oxidative stress, and apoptosis—that further exacerbate tissue damage and worsen neurological outcomes [11]. By directly affecting the upper and lower spinal cord pathways, any personal injury damages the blood–spinal cord barrier (BSCB), which in turn leads to a series of injuries such as spinal cord shock, generalized hypotension, vascular spasm, ischemia, and iatrogenic imbalances [12]. It is worth noting that secondary injuries can occur subsequent to a primary SCI. Overtime, these injuries can become aggravated or worsened. It includes cell death and inflammatory response, spinal cord edema, ischemia-reperfusion injury, neuronal and axonal degeneration, and scar tissue formation [13]. These secondary cascade reactions trigger the development of glial scarring and cystic cavities, which interfere with the normal process of myelin sheath formation and axonal regeneration and may lead to permanent damage [14]. Furthermore, the ischemic reperfusion injury that occurs following a SCI not only causes harm to endothelial cells and modifies in vivo vascular permeability [15], but also initiates the release of pro-inflammatory mediators, signaling proteins, reactive oxygen species, neurotoxic amino acids, and other toxins from activated microglial cells, astrocytes, neutrophils, and macrophages, thereby intensifying the inflammatory response [16]. Consequently, the treatment of SCIs should prioritize strategies to mitigate inflammation at the injury site and enhance axonal regeneration.

## 3. Exosomes Derived From Cells

Steroid therapy is commonly used in the management of SCI, primarily aimed at reducing inflammation and edema at the injury site, enhancing neuroprotection, and promoting tissue repair. It is most effective when administered during the acute phase of SCI to minimize secondary damage. Among the available steroids, methylprednisolone and dexamethasone are the most widely used, having demonstrated efficacy in reducing inflammation and limiting neuronal damage [17], have been a mainstay of clinical practice for an extended period. Furthermore, recent research indicates the potential benefits of estrogens and progestins in the management of SCIs [18]. While hormone administration early after injury can mitigate inflammation and prevent further damage, it also carries a number of risks. These treatments present a complex set of considerations, including an increased risk of infection and metabolic and neurological side effects, as well as potential osteoporosis and fracture risks associated with long-term use [19]. In this context, a number of innovative therapeutic approaches are being developed. Cellular therapies, in particular, appear to hold significant potential for neuroprotection and neuroregeneration in SCI. Various cellular transplantation strategies are currently under investigation for SCI treatment, including mesenchymal stromal cells, neural progenitor cells, oligodendrocyte precursor cells, Schwann cells, and olfactory ensheathing glia. These strategies appear to aim to enhance recovery and improve outcomes for patients with SCI. However, there are still immune rejection, risk of tumourigenesis, and ethical issues associated with such therapies [20]. In addition, it has been shown that mesenchymal stromal cells progressively lose their proliferative and differentiation potential during in vitro culture, which may lead to instability in therapeutic outcomes [21]. Therefore, we need to explore a safer and more effective approach for treating SCIs.

**Figure 2:**
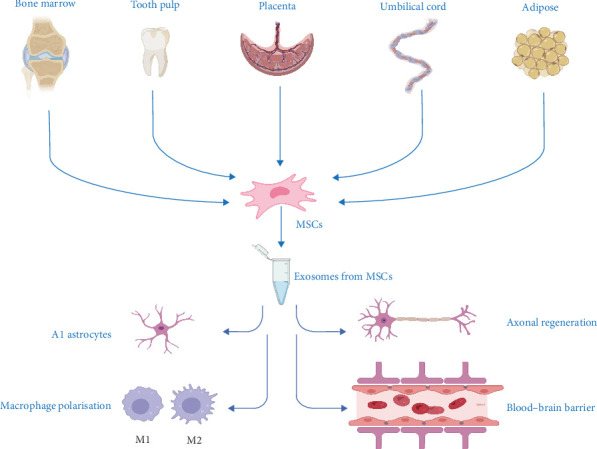
Therapeutic efficacy of exosomes from different MSCs in the treatment of SCI. MSCs can be obtained from bone marrow, dental pulp, umbilical cord, amniotic membrane, and adipose tissue. Exosomes derived from MSCs have anti-inflammatory and antiapoptotic effects and inhibit A1 astrocytes, promote axonal regeneration and macrophage polarization, and protect the blood–brain barrier. The image was produced by BioRender.com.

A number of studies have highlighted the crucial role of paracrine effects in the therapeutic processes of stem cell treatment for SCI [22]. Exosomes are small vesicles with membrane structures, ranging in diameter from approximately 30 to 150 nm. These vesicles are secreted by cells into the extracellular space via exocytosis and carry a diverse array of biomolecules, including nucleic acids, proteins, and lipids [23]. Compared to stem cell transplantation, exosomes offer several advantages: they have an extended retention time, minimal risk of causing cancer, high precision in targeting, low likelihood of immune rejection, and can readily penetrate the blood–brain barrier [24, 25]. Furthermore, studies have shown that exosome treatment significantly reduces cell death at the site of spinal cord damage. This intervention also substantially lowers the expression levels of cell death-promoting proteins and inflammatory mediators, such as TNF-α and IL-1β [26]. Another study found that exosomes can cross the blood–brain barrier and reach the injured spinal cord region, promoting axonal growth and neovascularization, while inhibiting microglia and astrocyte proliferation (Figure 2) [27]. Additionally, exosomal therapy reduces the tumorigenic risk linked to cell transplantation, providing a safer and more effective method by adjusting the microenvironment and delivering bioactive molecules.

The current challenges in translating exosomes to clinical practice primarily stem from the absence of standardized methods for isolation and purification. Differential centrifugation remains the most widely employed technique for isolating and concentrating exosomes. However, this method has yet to achieve complete separation of nonvesicular entities from exosomes. Additionally, there is a technological gap in efficiently producing and purifying exosomes on a large scale. In addition, quality control and standardization of exosomes is an important challenge for clinical translation. There is a lack of uniform quality standards and assessment methods, making it difficult to ensure the quality and stability of exosome products [28].

### 3.1. MSCs Derived Exosomes

MSCs are pluripotent stem cells with the capabilities of multidirectional differentiation and self-renewal. They can be obtained from diverse tissues such as bone marrow, adipose tissue, and human umbilical cord blood [29, 30]. Relative to other stem cell types, MSCs offer several advantages: they are easily isolated and preserved, exhibit rapid proliferation, and possess low immunogenicity. Currently, the primary methods for MSC transplantation include direct spinal cord injection, systemic administration via veins, and intrathecal injection [31]. While intravenous and intrathecal injections are less invasive, they suffer from low delivery efficiency to the injury site and inadequate therapeutic efficacy. Direct injection into the spinal cord, while enabling MSCs to be directly transplanted to the injury site, carries risks such as potential tissue damage, infection, and leakage of cerebrospinal fluid [32]. A key function of MSCs in tissue repair involves paracrine secretion, where exosomes are believed to play a crucial role [33, 34]. The inflammatory response following SCI is intricate, involving numerous interactions among cells and molecules, making outcomes unpredictable. Suppressing inflammation can mitigate the progression of tissue damage. Recent investigations have highlighted the robust anti-inflammatory effects of MSC-derived exosomes. These exosomes are shown to effectively reduce the expression levels of pro-inflammatory cytokines TNF-α, IL-1β, and IL-6, while also suppressing the activation of NLRP3 inflammasomes. Furthermore, they enhance the production of antiapoptotic protein Bcl-2 and anti-inflammatory cytokines such as IL-4 and IL-10 [35–37]. Furthermore, studies have demonstrated that MSC-derived exosomes administered intravenously efficiently target the SCI site, where they selectively bind to M2 macrophages [38]. These macrophages are known for their role in producing anti-inflammatory factors, thereby mitigating the inflammatory response at the injury site. More importantly, it has been shown that miRNAs in exosomes released from MSCs can be used to inhibit the activation of NLRP3 inflammatory vesicles. It has been shown that miRNA-133b released from MSCs can promote axonal growth [39]. Finally, it is worth mentioning that no study has yet reported the risk of tumourigenesis [40]. The higher safety and low immunogenicity of MSCs make them more appropriate for clinical use compared to stem cell transplantation.

#### 3.1.1. Exosomes Derived From Bone Marrow MSCs (BMSCs-Exos)

BMSCs exhibit neuroprotective properties and stimulate nerve and myelin regeneration while minimizing scarring, thereby facilitating spinal cord reconnection following injury [41]. This capacity holds promise for rebuilding the damaged spinal cord post-SCI. Pericytes, located in the basement membranes of microvessels throughout the body, including the spinal cord, are crucial mural cells. Extensive preclinical research indicates that pericytes serve as a pivotal component of the neurovascular unit (NVU), contributing significantly to various essential functions. The aforementioned roles encompass the regulation of the permeability of the BSCB, control of capillary flow, promotion of vascular growth and maintenance, removal of harmful substances, and regulation of immune cell entry into the central nervous system (CNS) [42–44]. Reduced numbers of pericytes promote the growth and maintenance of the vascular system and the elimination of harmful substances. A reduction in pericytes increases the permeability of the blood–brain barrier, disrupts the microcirculatory system and allows harmful blood components to infiltrate the CNS, further aggravating neurological dysfunction [45]. Zhou et al. [46] observed that BMSC-exos treatment significantly reduced neuronal apoptosis, promoted myelin rearrangement and significantly slowed down myelin desheathing, and increased the number of pericytes and endothelial cells in the vascular wall. Furthermore, it reversed blood–brain barrier leakage and inhibited the release of IL-1β, thus, facilitating the rehabilitation of motor abilities following SCI.

Following the onset of SCI, multiple pathological changes occur involving the phases of inflammation, proliferation, and remodeling. In the sequential transitions between these phases, monocyte-derived macrophages (MDMs) play a pivotal role in coordinating their actions through the release of a diverse array of signaling molecules [47]. During the early inflammatory phase, oligodendrocytes and neuronal cells undergo cell death, resulting in the buildup of substantial myelin remnants and cellular waste at the injury site. It is well known that myelin debris generated after SCI inhibits axonal regeneration and myelin regeneration, and in addition, these debris can stimulate inflammation progression [48]. Experiments by Wang et al. [49] demonstrated that these debris cause alterations in the M2 macrophage phenotype, and at the site of the lesion, macrophages that have lost their M2 phenotype and exhibit foam cell characteristics persist for a long period of time. These persistent macrophages have pro-inflammatory properties, exacerbate neurotoxicity, and impede the wound healing process [49]. Moreover, some clinical studies have found that it takes several years for myelin debris to clear from the site of injury [50]. BMSCs-Exos have been shown to enhance macrophage phagocytosis by up-regulating MARCO expression, a scavenger-like receptor predominantly expressed on macrophages. It promotes macrophage-mediated clearance of myelin debris, thereby alleviating associated inflammatory responses and aiding neurological recovery following SCI [51]. Type A1 astrocytes respond to injury by releasing inflammatory mediators such as IL-1α and TNF-α, which exacerbate the inflammatory response. These mediators recruit and activate additional immune cells, further intensifying the inflammatory environment [52]. In contrast, A1 astrocytes are activated through NF-κB signaling pathways in response to neuroinflammation [53]. Research has shown that BMSCs-Exos can decrease the prevalence of A1-type astrocytes following SCI. This effect may be due to the inhibition of NFκBp65 nuclear translocation, which in turn reduces inflammatory responses at the injury site [54]. In recent experiments, it was observed that the transplantation of BMSCs-Exos markedly reduced the levels of proapoptotic proteins (Bax) and inflammatory factors (IL-1β and MIP-1). Simultaneously, there was a substantial rise in the levels of anti-apoptotic proteins (Bcl-2) and inflammation-reducing cytokines [55]. These observations further validate the effectiveness of BMSCs-Exos in treating SCI through significant inflammation inhibition.

A multitude of studies have demonstrated that miRNAs are implicated in a plethora of biological processes and pathological mechanisms in SCI. These include neuroprotection and regeneration, inflammation regulation, glial cell activation, angiogenesis, and microenvironmental regulation [56, 57]. A study found that miR-29b overexpression led to decreased Bax expression and increased Bcl-2 levels, significantly reducing neuronal loss and promoting neuronal survival in the ventral horn of the gray matter, thus, enhancing neurological recovery after SCI [58]. Due to the inherent instability of miRNAs, efficient carrier systems are essential for their delivery. Exosomes are currently regarded as a highly promising vector for the efficient delivery of miRNAs to the CNS [59]. Exosomes of miRNA-29b-modified BMSCs have been shown to upregulate NF200 and GAP-43, markers of neuronal development and regeneration. These exosomes also reduce GFAP expression, thereby minimizing neuroglial scarring and promoting axonal regeneration. This effectively demonstrates that miRNA-29b-modified BMSCs exosomes can repair SCI [60]. Similarly, BMSCs-Exos modified with miRNA-494 have demonstrated the capability to lower the expression of GFAP, an astrocyte marker protein, while increasing neurofilament proteins [61]. Autophagy, a form of programmed cell death, involves a lysosome-dependent degradation pathway that helps maintain intracellular homeostasis. The latest studies show a clear link between boosting autophagy and restoring movement after SCI [62]. BMSCs-Exos have been demonstrated to significantly enhance LC3IIB levels in conjunction with Beclin-1, a protein marker of autophagy. This process is known to facilitate autophagosome formation [63]. Further studies demonstrated a significant upregulation of miR-21a-5p in BMSCs-Exos. This miRNA influences macrophage/microglia autophagy and pyroptosis by targeting the regulation of PELI1, ultimately aiding the recovery of motor function after SCI [64].

#### 3.1.2. Adipose-Derived MSC-Derived Exosomes (ADMSCs-Exos)

AD-MSCs and BM-MSCs share similarities in cell morphology and surface antigen expression, but exhibit differences in proliferation rates and multidirectional differentiation potential. Considering the higher concentration of somatic stem cells in adipose tissue compared to bone marrow, AD-MSCs are favored for research and clinical applications due to their abundant availability and ease of access [65, 66]. In a model, intravenous administration of exosomes from human epidural fat-derived MSCs (hEpi AD-MSCs) has demonstrated an ability to suppress the expression of several pro-inflammatory mediators. This suggests that hEpi AD-MSC-derived exosomes can effectively reduce the inflammatory reaction following SCI by modulating the levels of various signaling proteins [67]. It is of greater significance that exosomes derived from hypoxia-preconditioned ADSC fractions can facilitate the transition of M1-type microglia to M2-type by downregulating miR-130b-3p expression. This transition enhances the neuroprotective potential [68]. This may be related to the fact that the hypoxic environment induces exosomes to express certain specific targeting signals, thereby enhancing their migratory ability to damaged tissues or inflammatory regions [69]. Although hypoxia-treated exosomes theoretically exhibit a number of potential advantages, including enhanced bioactivity, optimized targeting properties, et cetera, the practical application of these theoretical advantages needs to be further validated and optimized through systematic experimental studies and exhaustive clinical trials. Nevertheless, the practical application of these theoretical advantages must be further verified and optimized through systematic experimental studies and detailed clinical trials.

#### 3.1.3. Exosomes Derived From Human Umbilical Cord MSCs (hucMSCs-Exos)

MSCs derived from umbilical tissue are notable for their high proliferative and self-renewal capacities. Their easy accessibility, low immunogenicity, and extensive differentiation potential have garnered significant attention in both scientific research and clinical applications. MSCs derived from the umbilical cord can substantially reduce the inflammatory response and oxidative stress by decreasing the release of pro-inflammatory cytokines such as IL-1β and IL-8 [70]. This, in turn, effectively inhibits apoptosis. Research has demonstrated that hucMSCs-Exos can counteract the LPS-induced increase in GFAP expression and decrease in NF200 levels. These exosomes effectively prevent apoptotic cell death through the inhibition of the TLR4 and NF-κB signaling pathway, thereby protecting neurons [71]. Furthermore, hucMSCs-Exos demonstrated the ability to reduce apoptosis and decrease inflammatory factor levels through the activation of the Wnt/β-catenin pathway. They also facilitated angiogenesis and axon growth while inhibiting astrocyte activation, which is crucial for promoting neuronal recovery [72]. In addition to inhibiting astrocyte activation, miR-146a-5p-modified hucMSCs-Exos also inhibited NF-κB pathway activation by targeting Irak1 and Traf6, which is a key component of neurotoxic astrocyte activation. Furthermore, miR-146a-5p-modified hucMSCs-Exos inhibited NF-κB pathway activation by targeting Irak1 and Traf6, which reduced neurotoxic astrocyte toxicity [73].

#### 3.1.4. Exosomes Derived From MSCs of the Human Placenta (hPMSCs-Exos)

The placenta is the tissue necessary for nurturing the development of the foetus, which includes placental membranes, chorionic villi, and amniotic membranes. These tissues are rich in mesenchymal stromal cells and stem cells can be obtained by specific isolation and culture methods. Due to their low immunogenicity, easier accessibility, relatively low cost, absence of ethical issues, and high proliferative capacity, they have many advantages as a novel stem cell–based therapeutic option for SCI treatment [74–76]. The results of the research indicate that hPMSCs-Exos significantly upregulated neural stem/progenitor cell markers, including SOX2, GFAP, PAX6, and Nestin. This evidence suggests that they have the potential to differentiate into new neurons in the damaged region of the spinal cord, which could be a promising avenue for the treatment of SCIs [77]. Additionally, hPMSCs-Exos promote the movement of human umbilical vein endothelial cells. Following SCI, there was a notable increase in vascular density, the proportion of vascular volume, and the network of vessels within the spinal cord. This reflects the positive role of hPMSCs-Exos in neural repair and regeneration [78]. More noteworthy is that recent experiments have suggested that hPMSCs-Exos combined with hyperbaric oxygenation can reduce the level of caspase-3 expression, and that the results of the combined treatment showed that oxidative stress and inflammation were suppressed [79]. This broadens a new path for our research on hPMSCs-Exos.

#### 3.1.5. Exosomes From Other MSC Sources

Besides the more commonly utilized MSCs-Exos, exosomes derived from dental pulp stem cells (DPSCs) can aid in SCI treatment. This is achieved through the inhibition of M1-type macrophage polarization, which is mediated by the ROS-MAPK-NF-κB P65 signaling pathway. The paucity of studies on DPSC-Exos for SCI has resulted in a lack of understanding of the mechanisms by which DPSCs-Exos promote neural and motor recovery in SCI models [80]. A recent study of human menstrual blood MSCs (MenSCs) combined with materials for the treatment of SCI found that implantation of exosome-coated biomaterials into the damaged area resulted in a reduction in inflammation, restoration of damaged neurons, and improvement in motor performance [81]. Although both MenSCs and BMSCs exhibit similar stem cell properties, MenSCs possess distinctive advantages. These include their relative ease of accessibility and non-invasive collection, their higher proliferation rate and longer lifespan, and so forth [82, 83]. There are no direct experiments that confirm the efficacy of MenSCs-derived exosomes in the treatment of nerve injury. However, based on the results of the aforementioned studies, it can be hypothesized that such exosomes have a significant and promising future in neurological recovery after injury.

### 3.2. NSCs-Derived Exosomes

Exosomes derived from nerve stem cells are rich in growth factors, miRNAs and proteins, and these active ingredients can promote neuronal growth, axonal reconstruction, and maintenance of vascular integrity, thus, helping to improve the function of damaged neural networks. In comparison to neural stem cell therapy for SCI, NSCs-Exos represents a safer therapeutic option. Exosomes are devoid of the risk of immune rejection and xenografts that may be caused by implanted cells. Furthermore, the bioactive components in exosomes have a wide range of regulating effects, which can play a comprehensive repairing and regulating role for neurons, astrocytes and immune cells in the surrounding environment. It was observed that VEGF-A in NSCs-Exos can promote angiogenesis and tissue healing in the spinal cord microvascular endothelium (SCMEC) and facilitate the recovery of motor function [84]. Another study demonstrated that miR-374-5p, which is highly expressed in NSCs-Exos, can promote autophagy and inhibit apoptosis in recipient cells by targeting SKT-4, thereby favoring the repair of SCI [85]. HDAC6, primarily localized in the cytoplasm, uniquely orchestrates the deacetylation of nonhistone substrates. This enzyme is crucial in various biological and disease-related mechanisms, including microtubule stabilization and apoptosis inhibition [86]. Hydrogels containing EGFRNSCs-Exos were applied directly to areas of SCI in animal models. The efficacy of these treatments was then assessed in terms of the restoration of motor function and the regeneration of neural axons. EGFRNSCs-Exos delivered miR-34a-5p to neurons, inhibiting the expression of HDAC6. This downregulation of HDAC6 enhanced microtubule stability, activating autophagic processes and promoting synaptic regeneration at the SCI site [87].

### 3.3. Schwann Cell-Derived Exosomes (SCDEs)

Schwann cells exhibit neuroprotective properties by clearing cellular debris and inflammatory factors around neurons. This action fosters a conducive setting for damaged regions and enhances nerve regeneration [88]. SCDEs exert anti-inflammatory influence and diminish neuronal cell death by suppressing M1 polarization and endorsing M2 polarization within macrophages/microglia. This modulation is likely associated with the control of MFG-E8 in SCDEs through the SOCS3/STAT3 signaling pathway in SCDEs [89]. Furthermore, SCDEs have been demonstrated to lower EGFR expression and inhibit the Akt/mTOR signaling pathway, thereby reducing neuronal apoptosis at the injury site. By enhancing autophagy, SCDEs have been shown to facilitate neural restoration after injury [90]. A recent study devised a nanofibre-based scaffolded hyaluronic acid (HA) hydrogel patch that releases SCDEs and methylprednisolone on the surface of injured nerves. The findings of the study indicated that the patch maintained the morphology of SCDEs and significantly restored motor function in SCI rats. This noninvasive drug-exosome dual release system material is promising for the treatment of SCI. It precisely inhibited inflammation after nerve injury by modulating the macrophage shift to the M2 phenotype and inhibited neuronal apoptosis by modulating TLR4/NF-κB, MAPK, and Akt/mTOR pathways [91].

### 3.4. Macrophage-Derived Exosomes

It is well established that macrophages exhibit two distinct phenotypes, designated M1 and M2. M1 macrophages secrete inflammatory markers such as IL-1β, TNF-α and IFN-γ, which exacerbate the injury. Conversely, M2 macrophages secrete anti-inflammatory cytokines and neurotrophic factors that contribute to a more favorable environment for neuronal survival and regeneration [92]. In a rat model of SCI, the injection of M2 macrophage exosomes through the tail vein resulted in higher scores on the BBB scale 14 days after the injury. Immunofluorescence staining and CD31 immunohistochemical analysis demonstrated that exosomes secreted by M2-type macrophages positively influence neural and vascular regeneration following SCI. The underlying mechanism may involve the HIF-1/VEGF signaling pathway [93]. The iTRAQ technique, which is used for the quantitative analysis of protein expression in samples, was employed to examine protein levels in M2-type macrophages and M2-derived exosomes. A sum of 307 proteins that were expressed differently were identified. The highest 20 upregulated proteins were examined using Gene Ontology (GO). Among these, OTULIN exhibited the most notable alterations, with its presence in M2-derived exosomes being significantly greater than in M2 macrophages. M2-derived exosomes play a role in vascular regeneration and neurological improvement after SCI by conveying OTULIN proteins and activating the Wnt/β-catenin signaling pathway [94]. Exosomes derived from peripheral macrophages have been demonstrated to block the PI3K/AKT/mTOR pathway. This results in microglial autophagy, which shifts the microglia towards an anti-inflammatory phenotype and reduces the inflammatory responses following SCI [95]. The above studies suggest that exosomes derived from macrophages have great potential to modulate inflammation and promote neuroregeneration. This approach presents a promising strategy for treating SCIs through various mechanisms.

### 3.5. Exosomes of Microglia Origin (MG-Exos)

Microglia are distributed throughout the white matter areas of both the brain and spinal cord, playing a crucial role in the CNS. Microglia can display different activation states, such as M1 and M2 types; M1 microglia mainly participate in inflammatory responses, while M2 microglia have anti-inflammatory and reparative functions and are crucial in regulating and repairing the neurological system [96]. Research revealed that administering exosomes obtained from M2 microglia (M2-EXOs) to an animal model of SCI improved motor function in mice suffering from spinal cord and nerve injuries. The mechanism is that M2-EXO inhibits the activation of neurotoxic A1 astrocytes, which is associated with inhibition of the NF-κB signaling pathway [97]. Moreover, MG-Exos showed notable beneficial effects on the development of blood vessels and the proliferation of cells in SCI models. MG-Exos prevent the activation of A1 astrocytes by stimulating the keap1/Nrf2/HO-1 pathway, protecting microvascular endothelial cells in the spinal cord from peroxide toxicity. This process supports blood vessel formation and enhances neurological function recovery after injury [98].

### 3.6. Exosomes of Other Cellular Origin

Within the spinal microenvironment, the NVU is essential for sustaining the blood–brain barrier. Pericytes, fundamental cells of the NVU, perform various roles including neuroprotection, maintaining stem cell pluripotency, and promoting angiogenesis [99, 100]. It has been demonstrated that pericyte-derived exosomes exert a beneficial effect on endothelial barrier integrity by inhibiting the JAK1/STAT3 pathway and, thereby improving endothelial barrier function and maintaining the stability of BSCB [101].

Although exosomes hold significant promise for treating spinal cord and nerve injuries, several challenges persist. Direct injection into the injury site can be hindered by local immune responses and the microenvironment, potentially impacting their survival and effectiveness. Additionally, large-scale production, storage, and transportation of exosomes remain difficult [102, 103]. Encapsulating exosomes in biomaterial scaffolds can preserve their biological activity, shield them from rapid degradation, and facilitate their prolonged release at the injury site. Biomaterial scaffolds are essential in nerve regeneration after SCIs. They provide guidance for nerve regrowth and efficiently deliver and anchor exosomes, cellular elements, or neurotrophic agents at the damaged site [104].

## 4. Different Types of Biological Material Scaffolds

Biomaterials for treating SCIs should possess excellent biocompatibility, biodegradability, controllable porosity, high safety, and other qualities [105]. Currently, biomaterial scaffolds can be classified into categories such as natural biomaterial scaffolds, synthetic biomaterial scaffolds, bioactive factor carrier scaffolds, cell carrier scaffolds, bioprinting scaffolds, and drug delivery scaffolds. The main components of the above scaffolds are mainly hydrogels, nanomaterials, biodegradable polymers, and 3D printing materials [106]. Additionally, a multitude of studies have demonstrated that biomaterials provide significant advantages for nerve function restoration and axonal regeneration after injury, showing great promise for SCI treatment applications [107].

### 4.1. Bioscaffolds Made of Hydrogel Materials

Hydrogel comprises a 3D interconnected polymer substance with a substantial amount of water content. Usually using water as a dispersing medium, it is able to bind well with the surrounding tissues and provide softness and elasticity similar to biological tissues, which is beneficial for supporting tissue growth and rehabilitation at the scene of damage; it can also gradually degrade and be metabolized by the surrounding tissues, avoiding the need for secondary surgery to remove the stent and reducing damage to the patient; it has excellent biocompatibility and degradability characteristics [108, 109]. In addition, hydrogels possess the ability to form scaffolds with 3D structures, providing superior support and spatial structure at the site of injury, which helps to promote cellular infiltration, proliferation, and differentiation [110]. Commonly, there are natural hydrogels, such as gelatin, sodium alginate, and chitosan as well as synthetic hydrogels including polyglycol and polyacrylic acid. In addition to this there are bioactive hydrogels, which can carry bioactive substances and have a promoting effect on tissue repair, such as gelatin–gelatinase complexes and collagen hydrogels [111].

Currently, the majority of SCI treatments utilize biocompatible polymers to mitigate subsequent inflammation [112]. These treatments also involve the creation of structured hydrogels or directional scaffolds to guide axonal growth, the adjustment of immune cell polarization, and the transplantation of cells at the injury site [113, 114]. Implantation of HA hydrogels in a rat model of SCI was effective in reducing neural scarring and maintaining axonal integrity, confirming a degree of neuroprotective effect of hydrogels as a base substrate for more complex scaffolds [115]. Building on this approach, we developed a composite hydrogel incorporating the KAFAK peptide and recombinant rat brain-derived neurotrophic factor (BDNF) with an injectable HAMC hydrogel. In a rat SCI model, this composite hydrogel released KAFAK and BDNF at the injury site, effectively reducing local inflammation while promoting neuronal survival and axonal regeneration [116]. In a separate study, it was demonstrated that treating SCI rats with a decellularized extracellular matrix (dECM) hydrogel encouraged macrophages in the damaged tissues to shift towards the M2 type. Moreover, a different research investigation demonstrated that macrophages in the injured tissues of SCI rats treated with dECM hydrogel were induced to polarize to the M2 type, aiding in the restoration of motor function following injury [117].

Recent advancements in cellular treatments for SCI have shown promising results. However, several unavoidable issues remain, the most pressing being the survival of transplanted cells at the injury site and the influence of the surrounding environment, which makes it challenging for the cells to persist and function over an extended period. To address these issues, stem cell-loaded hydrogel scaffolds have been developed [118]. Sodium thioredoxin-containing hydrogels encapsulating BMSCs not only reduce the production of oxidative stress markers in the SCI microenvironment, but also down-regulate pro-inflammatory cytokines, resulting in improved motor function [119]. A study using collagen matrices embedded with stem cells from bone marrow or umbilical cord MSCs for SCI treatment yielded positive results. Patients showed improved sensory and motor function and some regained the ability to walk independently postinjury [120]. This underscores the essential role of these effective scaffolds in the treatment protocols for SCI.

### 4.2. Nanomaterial Scaffolds

Nanoparticles, nanofibres, and nanosheets represent the primary types of nanomaterials employed as a means of treating SCI. These nanomaterials with specific functions are also known as functional nanomaterials. Functional nanomaterials treat SCI from the following aspects: direct protective effect of nerve cells, indirect neuroprotection by improving the microenvironment, stimulation of axons, and nerve regeneration [121–123]. Nanomaterials achieve indirect neuroprotection mainly by using them as drug carriers to efficiently deliver therapeutic drugs to damaged spinal cord tissue regions. In addition, certain nanomaterials have drug-like therapeutic effects and in vitro experiments have demonstrated that iron oxide nanoparticles attenuate oxidative stress with the potential to promote regeneration and axon growth [124, 125]. Selenium nanoparticles (SeNPs) have antioxidant activity, which can be used as a basis for designing superior and safe composite nanomaterials for SCI treatment [126]. The use of nanomaterials in SCI treatment has also been shown to reduce the risk of oxidation, which is a key factor in the development of a new drug. In excess of these functions, nanomaterials have also been shown to induce macrophage polarization at the site of injury and upregulate M2-type macrophage expression, indirectly protecting the nerves [127]. Importantly, several studies have pointed to nanomaterials guiding the regeneration of axons for substantial neurological recovery. The drug delivery platform of gold nanoparticles (AuNP) combined with polyethylene glycol enhanced axonal regeneration after injury [128]. Functional nanomaterials can target various facets of SCI for therapeutic purposes, potentially enhancing nerve repair and overall functional recovery. This offers new avenues for personalized treatment strategies for SCI.

### 4.3. Biodegradable Polymers

Biodegradable polymer scaffolds represent a significant advancement in biomaterial technology, playing a pivotal role across a range of applications from tissue engineering to medicine. These include poly(lactic acid) (PLA), poly(lactic acid-co-lactic acid) (PLGA), poly(L-lactic acid) (PLLA), polycaprolactone (PCL), among other materials [129]. Scaffolds prepared from such polymers exhibit the requisite mechanical properties and 3D structures, which can be employed to fill the SCI site and provide a supportive and regeneration-guided microenvironment. Furthermore, these scaffolds can be degraded gradually into harmless metabolites in vivo. Of particular significance is their potential role as a drug delivery system for the regulated release of therapeutic substances (such as drugs, growth factors, etc.) to achieve local treatment and promote tissue regeneration [130]. PLLA, a medical material approved by the FDA, has attracted considerable interest due to its affordability, simple manufacturing process, adaptable mechanical characteristics, and customizable surface features [131]. It has been demonstrated that 3D scaffolds with porous topologies prepared from PLLA materials provide bionic structures for neuronal cell adhesion and growth [132, 133]. As a consequence of the slow biodegradation properties of PLLA materials, they are often used as platforms for releasing drugs. Researchers designed and prepared a poly lactic acid (PLLA) porous film with longitudinal surface micropatterns and the presence of poly(anhydride) particles loaded with nerve growth factors on the surface of the material with controlled release properties and showed that this strategy enhanced the growth of nerve protrusions in PC12 cells [134]. Moreover, the PLLA scaffolds can be integrated with diverse therapeutic modalities, including electrotherapy, magnetotherapy, and autologous stem cell transplantation. These approaches have been the subject of investigation with regard to their potential for use in the reconstruction and regeneration of nerves [135, 136].

### 4.4. 3D Printing Materials

3D bioprinting is an advanced manufacturing technology that can produce complex and personalized biomimetic functional scaffolds with high precision and efficiency using biomaterial scaffolds, cells, and other bioactive molecules. And it has been effectively applied in bone, cartilage, peripheral nerves, cardiovascular system, skin, kidney, and other tissues or organs [56, 137, 138].

3D printing technology enables personalized manufacturing solutions for different individuals, ensuring that the printed tissue structure is optimally matched to the injury microenvironment to achieve the highest level of anatomical realism. Additionally, 3D printing can enhance axonal regeneration, supporting the formation of interaxonal synapses. This process aids in the restoration of neurological pathways within the spinal cord and the reestablishment of neural connections [139]. The techniques employed in the bioprinting of neural tissues have been classified into three principal categories: inkjet/droplet bioprinting, extrusion-based bioprinting, and laser-assisted bioprinting [140–142]. Of these techniques, extrusion bioprinting remains the most commonly employed [143]. Unlike inkjet printing, extrusion-based bioprinting employs continuous materials, enabling the creation of models with very high cell densities. Scaffolds with a high cell concentration offer a stronger support foundation for SCI recovery [144]. Compression-based bioprinting has been recognized as a key technology for advancing the treatment of nerve injuries. The utilization of extrusion bioprinting technology has yielded the fabrication of collagen/heparan sulfate scaffolds. The scaffolds exhibit a regular and porous internal structure and in vitro cultures have demonstrated their excellent biocompatibility with neural stem cells. Experiments have shown that implantation of such scaffolds provides continuous guiding pathways for axons and improves neurological function after SCI [145]. Similarly, another study utilized extrusion bioprinting to create a 3D collagen/silk protein scaffold (3D-CF) combined with NSCs. Experiments revealed that this scaffold, which replicated the distinctive shape of the spinal cord's gray matter, exhibited excellent biocompatibility. In addition to filling the damaged cavity, the regeneration of nerve fibers was observed, accompanied by an enhancement in the SCI rats' motor abilities [146]. To date, research into spinal cord bioprinting has focused on a narrow range of cell types and scaffold designs. Although certain multidimensional mesh structures are capable of imitating the linear conduction bundles of the spinal cord, these approaches are unable to fully replicate the complexity of the butterfly-shaped gray matter and its encircling white matter. Given the intricate organizational complexity and extensive neuronal pathways of the spinal cord, the engineering of bionic reconstructions represents a formidable challenge.

## 5. Exosomes Combined With Biological Scaffolds for the Treatment of SCI

A growing body of evidence has shown that combining exosomes with biological scaffolds holds great promise for treating SCI (Table 1) [81, 114, 147–150]. The bioactive molecules enriched in exosomes promote tissue regeneration and neuron protection, and the scaffolds provide a structure and guidance that help cell colonization and tissue regeneration. The field of exosome therapy presents a number of challenges when considered in isolation. One such challenge is the difficulty of sustaining the delivery of bioactive substances at the injury site. In order to enhance the efficacy of the treatment, a combination of approaches is required. Consequently, the use of exosome-infused biomaterial scaffolds has attracted significant attention.

**Figure 3:**
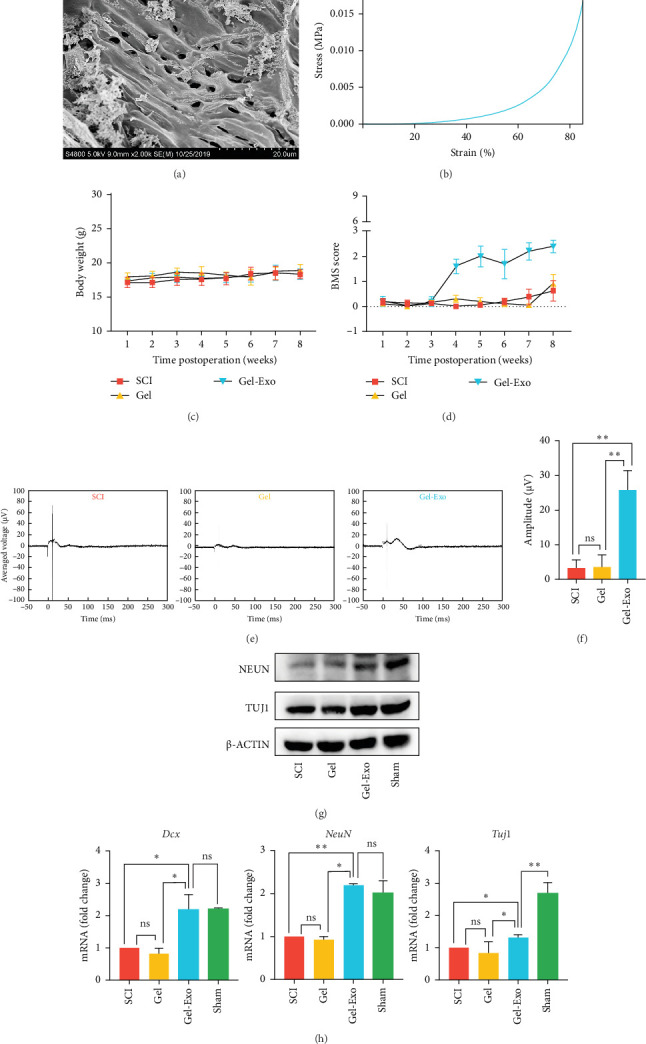
Gel-Exo facilitates recovery of complete SCI. (A) Gel-Exo SEM analysis. (B) Gel-Exo's representative stress–strain curve. (C) Postoperative weight changes. (D) BMS walking scores in the open field of mice with different treatments after SCI over the course of 8 weeks. *⁣*^*∗*^*p*  < 0.05 and *⁣*^*∗∗*^*p*  < 0.01 in comparison with SCI group. (E) Analysis of electrophysiology in mice under different treatments. (F) Amplitudes of MEPs: 3.23 ± 2.34, 3.52 ± 3.51, and 25.76 ± 5.65 μV for SCI, gel, and gel-Exo groups, respectively. *⁣*^*∗*^*p*  < 0.05 and *⁣*^*∗∗*^*p*  < 0.01, respectively, when compared with the SCI or Gel group. (G, H) Expression of major neuronal markers in injured spinal cord in each group. *⁣*^*∗*^*p*  < 0.05 and *⁣*^*∗∗*^*p*  < 0.01 when compared with SCI group or sham group. Adapted from He et al. [147 ]. Copyright 2022, Springer Nature.

In current studies, exosomes are mostly prepared from MSCs as MSCs secrete more exosomes compared to other cells [151]. BMSCs therapies are recognized for their immunomodulatory properties and have found application in treating severe inflammatory conditions. BMSCs-Exos supply different therapeutic performance factors and miRNAs that enhance neuronal regeneration and vascularization, decrease neuroinflammation and gliocytosis, and enhance motor and perceptual recovery following SCI [152]. Electroconductive hydrogel is a hydrogel scaffold combining a hydrophilic matrix, conductive polymer, metal nanoparticles, or conductive components such as carbon materials, which promotes neuronal differentiation of neural stem cells and guides synaptic elongation [153]. After implanting BMSCs-Exos immobilized in electroconductive hydrogel into the spinal cord hemisphere, the BMSCs-Exos demonstrated good bioactivity, which was sustained in the site of injury release and accumulation. The immune-regulating characteristics of BMSCs-Exos also helped polarize M2 microglia through the NF-κB pathway in the electroconductive hydrogel. Experiments in both live organisms and controlled laboratory environments have shown that the electroconductive hydrogel containing BMSCs-Exos activates the PTEN/PI3K/AKT/mTOR pathway [81]. This activation enhances the regeneration of neurons and the growth of axons. Conventional systemic administration could not accumulate enough exosomes at the injury site, so fibrin gels loaded with BMSCs-Exos were implanted into the injury site to achieve exosome immobilization at the lesion site, and these exosomes were found to contain a large amount of VGF, which was found to promote oligodendrocyte generation and facilitate the restoration of neurological function in the animal model of SCI (Figure 3) [147]. Since most SCIs are caused by out-of-hospital trauma, early intervention in the microenvironment at the site of injury is important for prognosis. This fibrin gel, prepared from a solution of fibrinogen and thrombin, will mimic the coagulation process and can be used as an emergency treatment for SCIs. It was observed that the fibrin gel containing MSCs-Exos decreased the inflammatory response postinjury and enhanced nerve regeneration [148]. This offers a novel approach for treating acute SCI with exosomes.

In a recent investigation, hPMSCs-Exos were encapsulated within a peptide-modified HA hydrogel (Exo-pGel) and transplanted at the site of injury. The exosomes were released in a sustained and effective manner, thereby improving the local microenvironment, reducing inflammation and oxidative response, and enhancing motorial recuperation in SCI rats' hindlimbs [114]. Similarly, another study employed alginate scaffolds in conjunction with hucMSCs-Exos for the treatment of pain resulting from L5/6 spinal nerve ligation (SNL). The findings indicated that this treatment effectively alleviated the pain associated with SNL and the anesthetic effect was still discernible on day 21 post-SNL [114]. The study also showed that the treatment was effective in relieving pain induced by SNL and the analgesic effect remained pronounced on day 21 post-SNL. Exosome miRNAs are treated as key components of functional molecules, which play an active role in inhibiting neuronal apoptosis. By immobilizing miR-138-modified hucMSCs-Exos in temperature-sensitive hydrogels, this novel delivery platform down-regulates the level of inflammation in BV-2 cells via the NLRP3-caspase1 signaling pathway and also downregulates intracellular reactive oxygen species levels to reduce neuronal apoptosis via the Nrf2-keap1 signaling cascade [149]. Given the structural complexity of the CNS, the progression of SCI extends beyond just inflammation-related pathways. Further studies are therefore essential to explore therapeutic strategies for neurological injury at various stages.

Beyond MSCs, exosomes from diverse cell types have been investigated for SCI therapy. Notably, local delivery of epidermal growth factor receptor-positive neural stem cell-derived exosomes (EGFR + NSCs - Exos) has been shown to effectively promote SCI repair. Through the integration of 3D printing technology with hydrogel scaffolds, researchers designed and fabricated a hydrogel-coated exosome patch for the treatment of SCIs. This innovative patch significantly enhanced nerve regeneration, highlighting its potential as a promising therapeutic strategy [87]. Exosomes derived from M2 microglia exhibit anti-inflammatory properties similar to those of M2 macrophages. In a study where these exosomes (M2-Exos) were immobilized within an electrically conductive hydrogel composed of tannic acid (TA) and polypyrrole (PPy), implantation of the hydrogel enhanced neurite and axon regeneration. Furthermore, the sustained release of M2-Exos not only reduced postinjury inflammatory responses but also inhibited the fibrotic response associated with hydrogel implantation [154].

## 6. Perspectives on the Use of Exosomes and Biological Scaffolds for Treating SCI

The functional components of stem cell-derived exosomes have been demonstrated to effectively promote neuronal growth, axonal extension, and synaptic reconstruction. The use of biomaterial scaffolds provides a means of enhancing the targeting of these exosomes in the vicinity of SCI, thereby optimizing the regenerative effect on the nerves. Exosomes derived from stem cells possess notable immune-regulating functions, which are further enhanced when combined with biomaterial scaffolds. This synergistic effect significantly promotes the repair of SCI. Nevertheless, a unified international standard for the isolation and refinement of exosomes, along with a consensus on the criteria for evaluating the security and curative potential of scaffolds, has yet to be established. Furthermore, the SCI model must be expanded to encompass a more comprehensive range of injuries to align it more closely with the actual clinical scenario.

## 7. Conclusion

Overall, the combination of exosomes and bioscaffolds holds great promise in treating SCI. This strategy can promote nerve regeneration, enhance cellular colonization and differentiation, exert immunomodulatory effects, and reduce scar formation. However, there are still some urgent problems that need to be solved, such as large-scale production and purification of exosomes, biocompatibility and degradation rate of biomaterial scaffolds, stability of exosomes, immune rejection, and individual differences. With the continuous advancement of technology, this combined treatment strategy is expected to achieve wider clinical applications in the future.

## Figures and Tables

**Table 1:** Recent studies on stem cell-derived exosome-bound biomaterials.

Biomaterial scaffolds	Cell-derived exosomes	Animal models	Results	References
GMP hydrogel	BMSCs-Exos	Right hemisection in mice	Inhibits inflammation, enhances NSCs recruitment, and promotes neuronal and myelin-related axonal regeneration	[81]
Fibrin gel	BMSCs-Exos	Transection in mice	Promote recovery of behavioral performance, electrophysiological performance, and neurogenesis	[147]
Fibrin glue	hucMSCs-Exos	Transection in rat	Reduces the oxidative and inflammatory microenvironment and promotes the restoration of urinary function	[148]
Alginate scaffolds	hucMSCs-Exos	Nerve root ligation in rat	Suppresses nerve inflammation and relieves pain caused by nerve damage	[114]
Temperature-sensitive hydrogel	hucMSCs-Exos	Contusion in rat	Inhibition of neuroinflammation leading to alteration of microglia activation and reduction of cellular oxidative damage	[149]
F127-polycitrate-polyethyleneimine hydrogel (FE)	ADMSCs-Exos	Transection in rat	Reduces inflammatory response and promotes tissue repair and motor function recovery	[150]

*Note:* We summarize recent studies on the use of exosomes combined with biomaterials for the treatment of SCI and report the findings, which indicate a positive effect on nerve regeneration.

## Data Availability

Data sharing is not applicable to this article as no datasets were generated or analyzed during the current study.
